# Maternal separation leads to regional hippocampal microglial activation and alters the behavior in the adolescence in a sex-specific manner

**DOI:** 10.1016/j.bbih.2020.100142

**Published:** 2020-09-19

**Authors:** S. Bachiller, A. Paulus, S. Vázquez-Reyes, I. García-Domínguez, T. Deierborg

**Affiliations:** a Experimental Neuroinflammation Laboratory, Experimental Medical Sciences, Lund University, Lund, Sweden; b Laboratorio de Fisiopatología Neurovascular, Centro Nacional de Investigaciones Cardiovasculares (CNIC), Madrid, Spain

**Keywords:** Maternal separation, Microglia, Adolescence, Neuroinflammation, Sex-differences

## Abstract

Early life adversities during childhood (such as maltreatment, abuse, neglect, or parental deprivation) may increase the vulnerability to cognitive disturbances and emotional disorders in both, adolescence and adulthood. Maternal separation (MS) is a widely used model to study stress-related changes in brain and behavior in rodents. In this study, we investigated the effect of MS (postnatal day 2–14, 3 ​h/day) in both, female and male adolescent mice. Specifically, we evaluated (i) the spatial working memory, anxiety and depressive-like behavior, (ii) the hippocampal synaptic gene expression, and (iii) the hippocampal neuroinflammatory response.

Our results show that MS significantly increased depressive-like behavior in adolescent female mice and altered the spatial memory in adolescent male mice. In addition, MS led to decreased expression of genes related to synaptic function (*5ht6r, Synaptophysin,* and *Cox-2*) and induced an exacerbated microglial activation in dentate gyrus (DG), CA1, and CA3. However, while the levels of hippocampal inflammatory cytokines were not modified by MS, they did follow a sex-specific expression in adolescent mice.

Taken together, our results suggest that MS induces long-term changes in hippocampal microglia and synaptic gene expression, alters the spatial memory, and induces depressive-like behavior in the adolescent mice, in a sex-specific manner.

## Highlights

In wildtype adolescent mice (6 weeks of age):

• Maternal separation alters spatial working memory in males and induces depressive-like behavior in females.
• Maternal separation changes hippocampal synaptic gene expression.
• Maternal separation activates microglia in dentate gyrus, CA1, and CA3 but does not affect hippocampal cytokine levels. However, males present higher levels of cytokines compared to females.

## 1 Introduction

Adolescence is a critical period for brain development when the brain undergoes different processes (including executive function development, synaptic stabilization and synaptic pruning (Selemon, 2013)) that induce profound emotional and cognitive changes. The hippocampus is a key brain region that regulates memory processes and emotions. Owing to its neuroanatomical connections and high expression of glucocorticoids and mineralocorticoids, the hippocampus is highly vulnerable to early environmental factors, such as stress or immune activation (Hoeijmakers et al., 2014). In fact, early life stress (ELS) exposure during childhood alters hippocampal development and/or its activation, which correlates with mood and memory disturbances found in human adolescents (Chugani et al., 2001; Carrion et al., 2010; Herringa et al., 2013) and animal models (Molet et al., 2014; Harrison et al., 2014).

Over recent years, neuroinflammation has emerged as a potential link between ELS and the emergence of neuropsychiatric disorders in adolescents. In the developing hippocampus, ELS alters microglia (proliferation, morphology and phagocytic activity (Johnson and Kaffman, 2018)) and can prime them, which has been associated with behavioral abnormalities later in life (Catale et al., 2020). Microglia also undergo sex-dependent maturation processes that affect, among other factors, cytokine release which might be linked to the differential response to ELS in adolescence (Schwarz and Bilbo, 2012; Grassi-Oliveira et al., 2016).

In this study, we hypothesized that the previously observed behavioral effects of ELS in adolescents (He et al., 2020) is mediated by increased brain inflammatory responses in mice. We used an established model of ELS, maternal separation (3 ​h/day, postnatal day 2–14). To study the effect of MS on the brain, specifically the hippocampal inflammatory response during adolescence, we examined: (i) the anxiety- and depressive-like behavior, and the spatial memory; (ii) the expression of genes involved in inflammation-induced depression and synaptic dysfunction in hippocampus (*Cox-2* (Muller, 2019), *5ht6r* (Rasenick, 2016) and *Synaptophysin* (Cui et al., 2020)); and (iii) the regional hippocampal microglia activation (in DG, CA1 and CA3) and the hippocampal cytokine concentrations in both female and male mice at 6 weeks of age.

## 2 Materials and methods

### 2.1 Animals

All the experiments were performed following the international guidelines on experimental animal research and approved by the Malmö-Lund Ethical Committee for Animal Research in Sweden (Dnr. 5.8. 18-01107/2018).

Four breeding cages with two C57bl/6 female mice bred with one C57bl/6 male mouse (9–12 weeks old) per cage were used in this study. Pups were weaned at P30, and age- and sex-matched wild-type littermates were group-housed (3-5 animals/cage) with bedding material, 12 ​h light/dark cycle, and water and food provided *ad libitum*.

### 2.2 Maternal separation

Maternal separation (MS) was performed as described by Teissier et al. (2020). Briefly, 7 female and 9 male pups were daily separated from their dams from postnatal days 2–14 (P2- P14), 3 ​h per day (09:00 a.m.-12:00 p.m.). MS pups were placed together into a clean cage with extra nesting material (cotton pieces) to keep them warm and with enough distance to avoid vocalized communication with their dams. After 3 ​h, pups were returned to their dams and kept undisturbed until the following day. Control litters (7 females and 7 males) were handled similarly to the MS pups from P2 to P14. At the end of the separation, there were no significant body weight differences between MS and control mice. At 6 weeks of age, when the experiment was concluded, there were body weight differences between sexes but not due to a MS effect.

### 2.3 Behavioral tests

#### 2.3.1 Elevated plus maze (EPM)

EPM was done to evaluate anxiety-like behavior. The mouse was gently placed in a closed arm facing the wall and it allowed to explore the maze for 5 ​min. The number of entries into each arm and the time spent in the open arms were recorded and used to calculate the anxiety index (AI) (Cohen et al., 2008):

$$AI=1-\left(\left(\frac{\;Time\;Open\;Arms\;}{Total\;time\;of\;the\;test}\right)+\left(\frac{Entries\;Open\;Arms}{\;Total\;number\;of\;entries\;}\right)\right)/2$$

#### 2.3.2 Tail suspension test (TST)

To assess depressive-like behavior, TST was performed. Mice were suspended 50 ​cm above the floor by the tail using adhesive tape, and the total time spent immobile during the 6 ​min of the test was quantified. Immobility was considered to be when the mice hung passively, not moving their limbs and body, and completely motionless.

#### 2.3.3 Y-maze test

The Y-maze test was done as previously described (Hansson et al., 2019). Mice were placed at the end of one arm facing the wall and allowed to explore the maze for 5 ​min. The number of entries was recorded, and the spontaneous alternation was defined as entries into the 3 arms on consecutive choices. The percentage of total alternations was represented.

### 2.4 Tissue processing

6-weeks-old mice were anaesthetized using isofluorane (5%) in oxygen (Virbac) and perfused transcardially with 0.9% saline solution. One hemisphere of the brain was fixed in 4% paraformaldehyde solution (Histolab) overnight (4 ​°C), and then stored in 30% sucrose solution for 48 ​h (4 ​°C). Coronal sections (40 ​μm) were obtained using a freezing microtome (Leica SM2000DR) and preserved in cryoprotective solution (30% sucrose [Sigma-Aldrich], 30% ethylene glycol [Sigma-Aldrich], 40% phosphate-buffered saline) at -20 ​°C.

Hippocampus was isolated from the other half of the brain and snap frozen at -80 ​°C until further RNA or protein isolation.

### 2.5 Immunofluorescence, image acquisition and image analysis

Immunofluorescence was performed as previously described (Bachiller et al., 2018). Free-floating coronal sections were permeabilized using Triton X-100 (Sigma-Aldrich) 1% (v/v) in PBS (PBS-T1%) for 1 ​h, incubated in the blocking solution (5% Normal Donkey Serum, PBS-T1%) for 1 ​h and then in primary antibody, anti-Iba1 (Wako, 1:500) at 4 ​°C overnight. Then, sections were rinsed for 1 ​h with PBS-T0.1% and incubated with the corresponding secondary antibody (1:500, donkey anti-rabbit 647, Invitrogen) for 1 ​h and finally mounted using ProLong Diamond Antifade Mountant (Invitrogen). Images were taken with a Nikon confocal A1RHD laser-scanning microscope using an 20X air objective with the same laser acquisition parameters and the same researcher, blinded to the groups. Analysis of the images was done using Fiji ImageJ software (W. Rasband, National Institutes of Health). Soma size analyses were performed as described by Hadar et al. (Hadar et al., 2017) in DG, CA1 and CA3. The image background was subtracted and then a threshold was manually set for microglial soma visualization and measurement. At least 2-3 brain sections (bregma -2.0 to -2.5 ​mm)/region/animal were analyzed.

### 2.6 RNA extraction and RT-qPCR analysis

Total hippocampal RNA was extracted using the TRI-reagent (Sigma-Aldrich) following the manufacturer’s instructions. Quantitative RT-qPCR analysis (CFX96™ Real-Time System-C1000™ Thermal Cycler, BioRad) were performed for the following genes (5′-3′): *5ht6r* (Forward: CTTCCTGCTATGCTTGGTGGT; Reverse: TGTTAGGGTTGAGGTTCAGTCT); *Synaptophysin (Syp)* (Forward: TCTTTGTCACCGTGGCTGTGTT; Reverse: TCCCTCAGTTCCTTGCATGTGT); and *Cox-2* (Forward: CCAGCACTTCACCCATCAGTT; Reverse: ACCCAGGTCCTCGCTTATGA). Relative gene expression was represented as ΔCt method, normalized to the expression of *Actin* as the housekeeping gene.

### 2.7 Protein extraction and Meso scale

To measure the cytokine concentrations in the hippocampus, a Meso Scale Discovery V-Plex Plus Kit (MSD Mesoscale Discovery, USA) for proinflammatory mouse markers was performed as previously described (Boza-Serrano et al., 2019). The detection ranges were the following: IL1β (1670–0.408 ​pg/mL), IL10 (3410–0.833 ​pg/mL), IL5 (967–0.236 ​pg/mL). The concentrations for TNF-α, IFN-γ, IL12, IL2, IL4, IL6 and KC/GRO, were below the detection range and excluded from the study.

### 2.8 Statistical analysis

All statistical analyses were performed using GraphPad Prism 8.0 Software for Macintosh (GraphPad Software, San Diego, CA, USA). For the behavioral experiments, at least 7 animals/group were included. Otherwise, at least 4 animals/group were analyzed. We used different unpaired statistical analysis based on the results of the normality and lognormality test (Shapiro-Wilk test). If data was normally distributed, two-way ANOVA followed by Tukey’s test for multiple comparisons was performed. For non-parametrically distributed data, Kruskal-Wallis followed by Dunn’s test for multiple comparisons was used. Data is reported as mean ​± ​SD. P values ​≤ ​0.05 were considered statistically significant and are stated in the figure legends.

## 3 Results

### 3.1 MS mice display depressive-like behavior and spatial memory impairment in a sex-specific manner

**Fig. 1:**
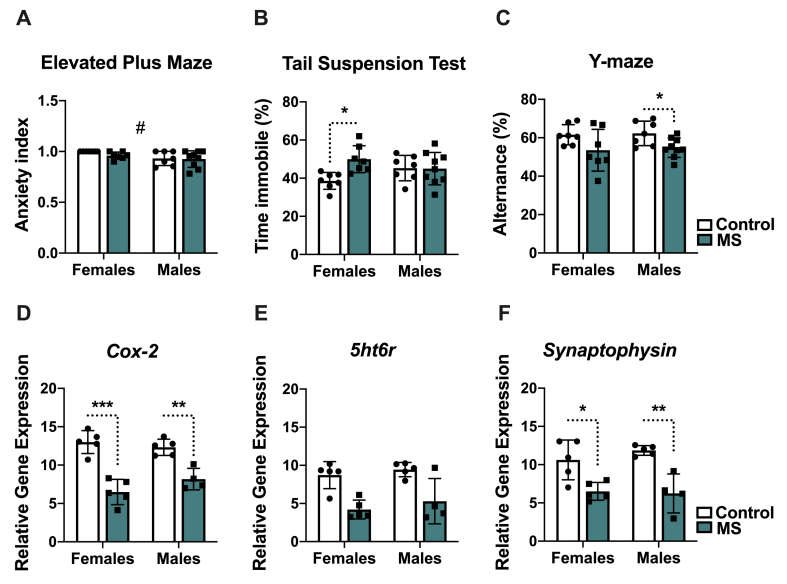
**Maternal separation (MS) alters behavioral outcomes and hippocampal gene expression in the adolescents (6 weeks old) in a sex-specific manner**. (A) Anxiety-like behavior was not induced by MS. However, females presented a higher AI than male mice. (B) Depressive-like behavior was elevated in MS females as revealed by the tail suspension test. This effect was not observed in MS males. Contrarily, MS males presented a spatial working memory impairment as shown by the decrease in spontaneous alternation in the Y-maze compared to controls (C). MS mice presented a significant decrease in hippocampal relative gene expression of *Cox-2* (D)*, and Synaptophysin* (F). While not significant differences were not found for the expression of *5ht6r* (E), a trend towards decreased expression was also observed in MS mice compared to controls. Behavioral tests: Controls (Females, n ​= ​7; Males, n ​= ​7); MS (Females, n ​= ​7; Males, n ​= ​9); Gene expression: Controls (Females, n ​= ​5; Males, n ​= ​5); MS (Females, n ​= ​5; Males, n ​= ​4). ∗ statistical differences between MS and their controls; # statistical differences between female and male mice. Data are shown as mean ​± ​SD. ∗p ​< ​0.05; ∗∗p ​< ​0.01; ∗∗∗p ​< ​0.001.

Anxiety-like behavior was evaluated using EPM. Our results did not show an effect from MS in the AI (F (1,26) ​= ​1.14; p ​= ​0.2947) (Fig. 1A) between the MS mice and controls (females: p ​= ​0.6162; males: p ​= ​0.9938) [time/entries in open arms (Average ​± ​SD): Females: Ctrl (0/0); MS (2.17 ​± ​3.52/1.14 ​± ​1.34); Males: Ctrl (7.78 ​± ​9.18/2.28 ​± ​2.75); MS (3.43 ​± ​4.05/2.44 ​± ​1.79)]. However, females presented a significantly higher AI compared to males (Sex effect: F (1,26) ​= ​5.47; p ​= ​0.0273).

Using TST, we assessed depressive-like behavior (MS effect: F (1,26) ​= ​4.69; p ​= ​0.0397). Compared to controls, MS females had a significant increase in the percentage of immobility time (p ​= ​0.0249). However, no differences were found between MS males and controls (p ​= ​0.999) (Fig. 1B).

We also evaluated the spatial memory using the Y-maze test (Fig. 1C). Our data showed an overall effect of MS (F (1,26) ​= ​8.75; p ​= ​0.0065) and a significant decrease in spontaneous alternation in the MS male group compared to controls (p ​= ​0.0204) but no differences in MS females (p ​= ​0.1751).

### 3.2 MS alters hippocampal expression of synaptic plasticity genes

The relative hippocampal gene expression of *Cox-2* (F (1,15) ​= ​66.5; p ​< ​0.0001) (Fig. 1D), *5ht6r* (H ​= ​9.562; p ​= ​0.0227) (Fig. 1E), and *Syp* (F (1,15) ​= ​31.3; p ​< ​0.0001) (Fig. 1F) was significantly reduced in the MS mice. Compared with their controls, significant statistical differences at the gene expression level were found for *Cox-2* (females: p ​< ​0.0001; males: p ​= ​0.0028) and *Syp* (females: p ​= ​0.0169; males: p ​= ​0.0024). While no significant differences were found for *5ht6r* gene expression levels (females: p ​= ​0.1475; males: p ​= ​0.2998), there was an evident trend towards decreased expression in the MS groups.

### 3.3 MS enhances microglial activation in DG, CA1 and CA3 following a sex-specific pattern but does not alter the hippocampal cytokine concentrations

**Fig. 2:**
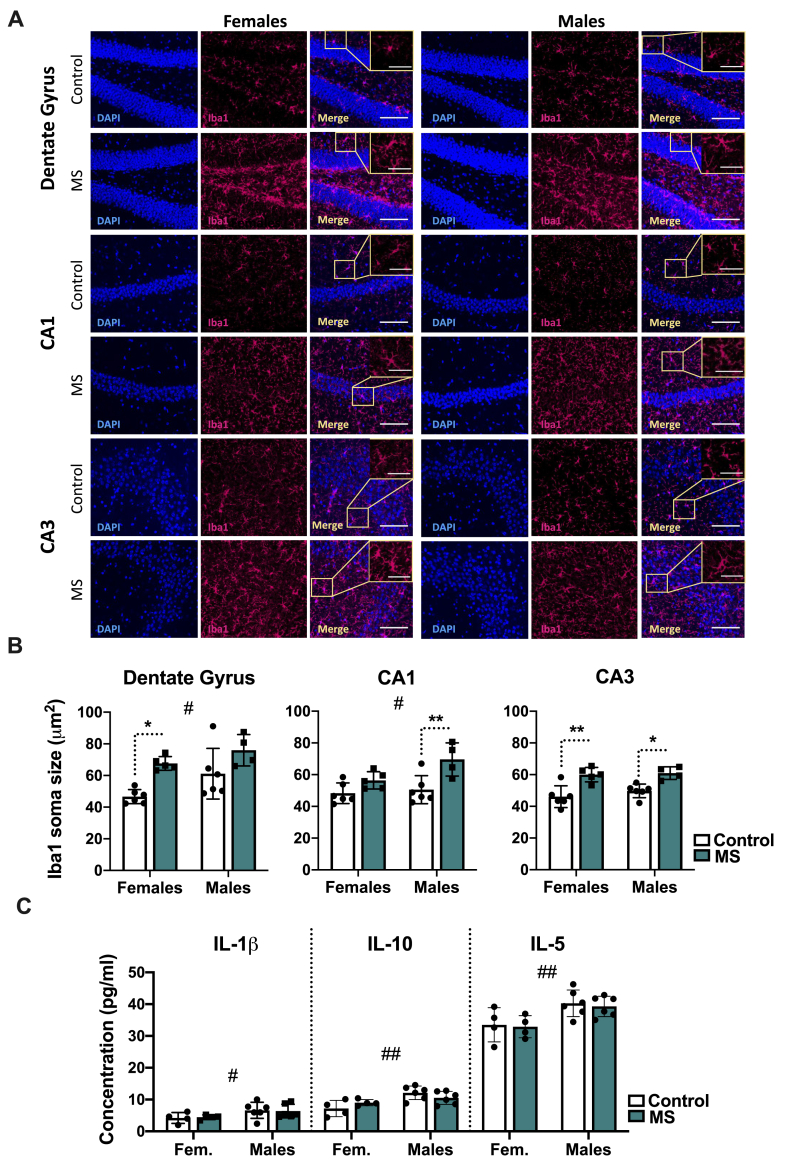
Maternal separation (MS) induces regional hippocampal microglial activation in DG, CA1 and CA3 in adolescents but does not alter the cytokine concentration. (A) Microphotographs of different hippocampal sections: dentate gyrus, 22 CA1 and CA3 (Bregma - 23 2.00 to -2.5 ​mm, 20X magnification). Inserts in the upper right of the merge images show different microglial cells at higher magnification. (B) MS induced a significant microglial activation in dentate gyrus, CA1 and CA3, measured as an increase in Iba1 soma area. Specifically, females’ dentate gyrus (left), males’ CA1 (middle) and both sexes’ CA3 (right) were activated by MS compared to controls. Furthermore, in DG and CA1 we observed a sex dependent activation, that was higher in male compared to female mice. (C) MesoScale assay for hippocampal cytokine quantifications revealed significantly increased cytokine levels in males but no differences between groups due to MS. Scale bars: 100 ​μm. Scale bar inserts: 50 ​μm. Image analysis: Controls (Females, n ​= ​6; Males, n ​= ​5); MS (Females, n ​= ​6; Males, n ​= ​4); Cytokine quantifications: Controls (Females, n ​= ​4; Males, n ​= ​4); MS (Females, n ​= ​6; Males, n ​= ​6). ∗ statistical differences between MS and their controls; # statistical differences between female and male mice. Data are shown as mean ​± ​SD. ∗p ​< ​0.05; ∗∗p ​< ​0.01.

Iba1 soma size measurements were performed in DG, CA1 and CA3 to evaluate the regional hippocampal microglial activation (Fig. 2A). Our results showed that MS induces microglial activation in DG (F (1,17) ​= ​15.9; p ​= ​0.001), CA1 (F (1,17) ​= ​15.3; p ​= ​0.0011), and CA3 (F (1,17) ​= ​30.0; p ​< ​0.0001). Regional activation also differed by sex in DG (F (1,17) ​= ​6.46; p ​= ​0.0211) and CA1 (F (1,17) ​= ​4.93; p ​= ​0.0403) but not CA3 (F (1,17) ​= ​0.987; p ​= ​0.3345). In DG, MS females showed increased microglial soma size (p ​= ​0.0159) compared to controls. However, no such difference was found in males (p ​= ​0.1453). In CA1, we observed the opposite effect of MS as soma size was significantly increased in the male group (p ​= ​0.0077) and no differences was seen in the female groups (p ​= ​0.3514). On the other hand, CA3 seems to be the most affected hippocampal region by MS in all the groups compared to controls (females: p ​= ​0.0019; males: p ​= ​0.0183).

Cytokines were measured in hippocampus using a Meso Scale Discovery assay (Fig. 2C). At 6 weeks of age, we did not find a significant effect of MS on IL-10 (F (1,16) ​= ​0.0234; p ​= ​0.8802), IL-1β (F (1,16) ​= ​0.129; p ​= ​0.7239), and IL-5 (F (1,16) ​= ​0.180; p ​= ​0.6768) levels. However, the cytokine concentrations significantly differed between sexes, with an overall higher levels in males of IL-10 (F (1,16) ​= ​12.0; p ​= ​0.0032), IL-1β (F (1,16) ​= ​4.63; p ​= ​0.0471); and, IL-5 (F (1,16) ​= ​12.6; p ​= ​0.0027).

## 4 Discussion

Adolescence is a particularly important period when cognitive functions undergo final development and disturbances in the emotional regulation processes potentially predispose the individual to psychiatric disorders.

Our results showed no MS effect on anxiety, whereas depressive-like behavior was increased in MS females but not MS males. Similar results have been described in adolescent female rats but not male rats exposed to chronic social stress (McCormick et al., 2008). However, inconsistent data has been published regarding MS and anxiety- and depressive-like behavior in adolescents (Shin et al., 2016; Banqueri et al., 2017). This can be explained by (i) the use of different MS protocols, which can affect different stages of the brain development; (ii) the lack of comparisons between sexes; (iii) the strain of rodents; and (iv) the context-dependent variability in these behavioral tests.

Nevertheless, our spatial memory results showed an impairment in MS males but not MS females, which is consistent with previous reports (do Prado et al., 2016; Brenhouse and Andersen, 2011). Moreover, it has been demonstrated that the exposure to MS in male juvenile rats but not female juvenile rats alters hippocampal-prefrontal cortex networks, which correlates with spatial memory dysfunction (Reincke and Hanganu-Opatz, 2017). Altogether, our behavioral results reveal a sex-dependent effect. Similar sex-dependent results have also appeared in adolescent human studies where the exposure to ELS contributes to hippocampal dysfunction with deficits in episodic memory and the development of depression (Barch et al., 2019; Colle et al., 2017).

In hippocampus, ELS alters dendritic branching and synaptic plasticity, and decreases hippocampal volume in both adolescents and adults (Vythilingam et al., 2002; McEwen, 2000; van der Kooij et al., 2015). *Syp* expression is closely related to synaptic plasticity in hippocampus, and downregulation of this protein has been found to be associated with stress-induced depressive-like behavior in rodent models (Reines et al., 2008; Thome et al., 2001) independently of sex (Cui et al., 2020). Likewise, *5ht6r* is a widely and highly expressed gene in cognitive regions (Woolley et al., 2004), and its pharmacological inhibition enhances glutamatergic neurotransmission in hippocampus (Dawson et al., 2001), suggesting a role in memory processes. On the other hand, *Cox-2* is localized to glutamatergic neurons, and its inhibition alters spatial memory as measured by the Water Morris Maze Test in rats (Teather et al., 2002). Hence, we speculate that the downregulation of *5ht6r*, *Syp* and *Cox-2* found in this study might be due to postnatal MS exposition and, in the long-term, alters the spatial memory in adolescent male mice. However, the effects of MS on hippocampal neurobiology in females remain to be determined.

Microglia are central in shaping/pruning neural networks during development. In the adult mouse hippocampus, microglia follow a regional distribution (Choi and Won, 2011; Long et al., 1998), suggesting a role in the regional-specific vulnerability of this area (Jinno et al., 2007). Therefore, we evaluated if the microglial activation followed a region- and sex-specific pattern in the hippocampus of MS adolescent mice. Our results revealed higher microglial activation in the MS mice in DG, CA1 and CA3. Moreover, our analysis showed a sex-dependent difference in microglial activation in DG and CA1 but not in CA3. Anatomically, the hippocampus is affected differentially by ELS; for example, in DG, ELS alters neurogenesis, decreases the number of granule cells (Kozareva et al., 2019), and induces an exacerbated glial activation (Diz-Chaves et al., 2012, 2013; Reus et al., 2017), whereas in CA1 and CA3, it alters the LTP and the dendritic branching of pyramidal neurons (Shin et al., 2016; Heydari et al., 2019). In our study, the largest effect of MS on microglia activation was found in CA3, a hippocampal region that is crucial to the stress response and that acts as a link to stress-induced neuronal plasticity and memory function (Lisman, 1999). These changes suggest that microglia could be participate in the synaptic remodeling even during adolescence.

On the other hand, the hippocampal cytokine levels were not affected by MS, but sex-differences were observed with overall higher concentrations in male mice. Growing evidence suggests that MS produces long-term effects on the immune system (Wieck et al., 2013; Danese et al., 2007), but only few studies address the importance of sex (Grassi-Oliveira et al., 2016; Avitsur et al., 2013). In adolescent rats, maternal deprivation induced an increase of TNF-α and IL6 in the hippocampus in both sexes, but those authors did not find differences in the IL10 (Stroher et al., 2020), consistent with our results. Contrarily to our results, Grassi-Oliviera et al. (Grassi-Oliveira et al., 2016) described a sex-dependent increase of inflammation in adolescent MS rats. However, they analyzed peripheral cytokines instead of hippocampal ones, and additionally, they looked at different ages compared to our study. Still, unpublished results from our lab show that, in adult mice, MS induces differential cytokine release both peripherally and in the hippocampus, suggesting that age might be an important factor to consider in these approaches.

The developing male brain has more microglia and mast cells and higher levels of inflammatory molecules (Lenz et al., 2018), which may contribute to the sex-related differences observed in the stress response of adolescents and adults (Meagher et al., 2010; Rana et al., 2012). Recently, Speirs and Tronson (2018) have shown sex differences in hippocampal cytokines after LPS administration in adult mice (bioRxiv, non-peer reviewed). They describe a differential cytokine response to LPS with a faster response in females than in males. We speculate that the lack of differences in the hippocampal cytokines between our MS mice and controls might be explained by the age of analysis as the cytokine activation may already have been resolved. Likewise, since the resolution may be faster in females than in males, this could explain the reduction of cytokines in females compared to the overall increase in males.

In summary, this study found that MS long-term strongly impacts changes in hippocampal microglia activation and synaptic gene expression in the adolescence. Also, sex plays a crucial role in the ELS-induced behavioral variations highlighting the significance of including males and females in the analyses to achieve better translational results. Further studies are needed to understand how ELS can affect the male and female brain differently and how this pathogenesis contribute to psychiatric diseases.

## Funding

This work was supported by the Olle Engkvist Foundation, the Strategic Research Area MultiPark at Lund University, the 10.13039/501100005753Royal Physiographic Society, the Swedish Alzheimer Foundation, the Swedish Brain Foundation, the 10.13039/501100003173Crafoord Foundation, the A.E. Berger Foundation, the Swedish Parkinson Foundation and the 10.13039/501100006310Swedish Medical Research Council.

## Declaration of competing interest

None.
